# Complex Odor from Plants under Attack: Herbivore's Enemies React to the Whole, Not Its Parts

**DOI:** 10.1371/journal.pone.0021742

**Published:** 2011-07-13

**Authors:** Michiel van Wijk, Paulien J. A. de Bruijn, Maurice W. Sabelis

**Affiliations:** University of Amsterdam, Institute for Biodiversity and Ecosystem Dynamics (IBED), Section Population Biology, Amsterdam, The Netherlands; University of Utah, United States of America

## Abstract

**Background:**

Insect herbivory induces plant odors that attract herbivores' natural enemies. Assuming this attraction emerges from individual compounds, genetic control over odor emission of crops may provide a rationale for manipulating the distribution of predators used for pest control. However, studies on odor perception in vertebrates and invertebrates suggest that olfactory information processing of mixtures results in odor percepts that are a synthetic whole and not a set of components that could function as recognizable individual attractants. Here, we ask if predators respond to herbivore-induced attractants in odor mixtures or to odor mixture as a whole.

**Methodology/Principal Findings:**

We studied a system consisting of Lima bean, the herbivorous mite *Tetranychus urticae* and the predatory mite *Phytoseiulus persimilis*. We found that four herbivore-induced bean volatiles are not attractive in pure form while a fifth, methyl salicylate (MeSA), is. Several reduced mixtures deficient in one component compared to the full spider-mite induced blend were not attractive despite the presence of MeSA indicating that the predators cannot detect this component in these odor mixtures. A mixture of all five HIPV is most attractive, when offered together with the non-induced odor of Lima bean. Odors that elicit no response in their pure form were essential components of the attractive mixture.

**Conclusions/Significance:**

We conclude that the predatory mites perceive odors as a synthetic whole and that the hypothesis that predatory mites recognize attractive HIPV in odor mixtures is unsupported.

## Introduction

Since the discovery that plants release herbivore-induced plant volatiles (HIPV) and thereby recruit predatory arthropods, researchers have sought ways to harness this chemical communication system. This led to a search for individual HIPV that act as predator attractants [1]. If predatory arthropods perceive odor mixtures as collections of classifiable chemical components that function as “attractant” or “repellent” it should be possible to manipulate the distribution of predatory arthropods in the environment through manipulation of HIPV. This possibility gained support from experiments wherein transgenic plants that constitutively produced (3S)-(E)-nerolidol were preferred by predators over non-transgenic control plants [2]. Because many herbivore induced compounds have been found to be attractive to predators the predominant line of thinking has become that only the induced attractants are important in indirect defence. There are currently many research programs that attempt to improve biocontrol through either the addition of synthetic attractants (HIPV) to crops or through the production of transgenic crops that constitutively produce novel HIPV.

The notion that an odor mixture is perceived as a collection of components that can be classified as “attractants” and “repellents” is however challenged by current ideas about the perception of olfactory information. For both arthropods and vertebrates, it has been suggested that odor mixtures are not perceived as a collection of individual components but rather as a synthetic whole [3], [4]. The black bean aphid *Aphis fabae* is repelled by nine host-plant compounds while a mixture of these is an attractant [5]. The parasitoid *Cotesia vestalis* is attracted to a mixture 4 HIPV presented against a background of clean cabbage odors whereas none of the components of this mixture acts as an attractant [6]. The hawk moth *Manduca sexta* does not respond to the individual components of an attractive floral odor while a blend of these components elicits strong attraction [7]. There is, however, also evidence supporting the elemental perception of food odors. Vinegar is a component of rotting fruit and *Drosophila melanogaster* is innately attracted to vinegar [8]. Thus, it appears that an element of food-associated odors mediates innate attraction in fruit flies in much the same way as HIPV are thought to mediate predator attraction. The earlier examples, however, suggest that odor mixtures are rather perceived as a synthetic whole and not as a collection of functional components.

If odors are perceived as a synthetic whole, the components of an odor mixture may no longer be recognizable. This difference in the ability to perceive components in odor mixtures between elemental- and synthetic perception of odors can be understood in the following way. Consider how two odors that have many components in common such as the odors of an infested and a non-infested plant can be discriminated. If odors are perceived as elemental objects the neuronal representation of one odor will largely overlap with the representation of the other. This has to be so, because only then the same component elicits the same neuronal activity in both odors thereby enabling recognition. Because of this constraint the synaptic changes that result from learning about one odor will also affect synapses activated by the other. Also, the more complex the odor, the greater the ensemble of neurons representing it. The representational constraint can be avoided if odors are perceived as a perceptual whole. The correlated olfactory input elicited by similar odors can be decorrelated and depending on the degree of decorrelation, the representations of each odor need not overlap. Components may then however no longer be recognizable as parts of odor mixtures.

Here, we address the question whether herbivore-induced components in the odor of herbivore-infested plants, or the odor mixture as a whole, function as predator attractants. The study system consists of the predatory mite *Phytoseiulus persimilis* Athias-Henriot which exclusively relies on chemical signals emitted by plants to locate distant patches of herbivorous mites, being their prey [9]. Under natural conditions *P. persimilis* predominately feeds on herbivorous mites such as the two-spotted spider mite *Tetranychus urticae* Koch. Plants emit HIPV upon infestation by *T. urticae* [10], [11], which makes the plant attractive to predatory mites [9], [12]. Spider mites are highly polyphagous [13] and the quantitative and qualitative release of spider-mite-induced volatiles varies with plant species [14]. *Phytoseiulus persimilis* copes with this variability in spider-mite-induced plant odors by learning from experience. Olfactory preference is acquired during development and through associative learning in the adult phase [15], [16], [17], [18], [19].

Whereas experience modulates olfactory preference in *P. persimilis*, several lines of evidence suggest that the HIPV part of odor mixtures functions as an attractant to this predator. First, *P. persimilis* often prefers the odor of spider-mite infested plants over conspecific control plants even though it lacks experience with the specific odor mixtures [20]. Second, typical spider-mite induced plant volatiles can be attractants [2], [11], [19], [20]. Third, transgenic expression of HIPV in strawberry made these plants more attractive to *P. persimilis* than control plants [2]. On the other hand several lines of evidence contradict a special status of HIPV. Attraction to HIPV in their pure form is weak compared to attraction to the full blend of spider-mite induced plants [19]. The chance of finding predatory mite attractants among typical spider-mite-induced plant volatiles is not greater than finding them among compounds not associated with spider mite herbivory [19]. In odor mixtures the response to the whole appears to be more than the sum of its parts. For example, two components that elicit no response in their pure form may, as a binary odor mixture elicit a strong response [21]. Finally, HIPV does not appear to have a specific role in predator attraction since *P. persimilis* acquires a preference for control plants over HIPV producing plants just as readily as the reverse [17], [19].

To experimentally address the question whether predatory mite attraction to odors from herbivore-infested plants results from attraction to HIPV or from attraction to the mixture as a whole, we created an artificial odor mixture that mimicked the odor of spider-mite-infested Lima bean so well that the mites did not discriminate between the two. Upon spider mite infestation, Lima bean predominately produces the following five HIPV that were all part of the artificial mixture: methyl salicylate (MeSA), β-ocimene, cis-3-hexenyl acetate, (E)-4,8-dimethyl-1,3,7-nonatriene (DMNT) and (E,E)-4,8,12-trimethyl-1,3,7,11-tridecatetraene, (TMTT) [22], [23]. *Phytoseiulus persimilis* was cultured on spider-mite-infested Lima bean, thereby ensuring that the mites acquired a preference for this odor. For each of the HIPV we asked the following two questions: (1) What is the attraction of *P. persimilis* to the individual component, (2) What is the attraction of *P. persimilis* to the full mixture compared to a mixture lacking a particular component. To assess whether the non-induced part of the odor of herbivore infested plants contributes to the attraction of predatory mites, these experiments were performed both in the absence -, and in the presence of the odor of non-infested Lima bean.

## Results

### Response to the artificial mixture

**Figure 1:**
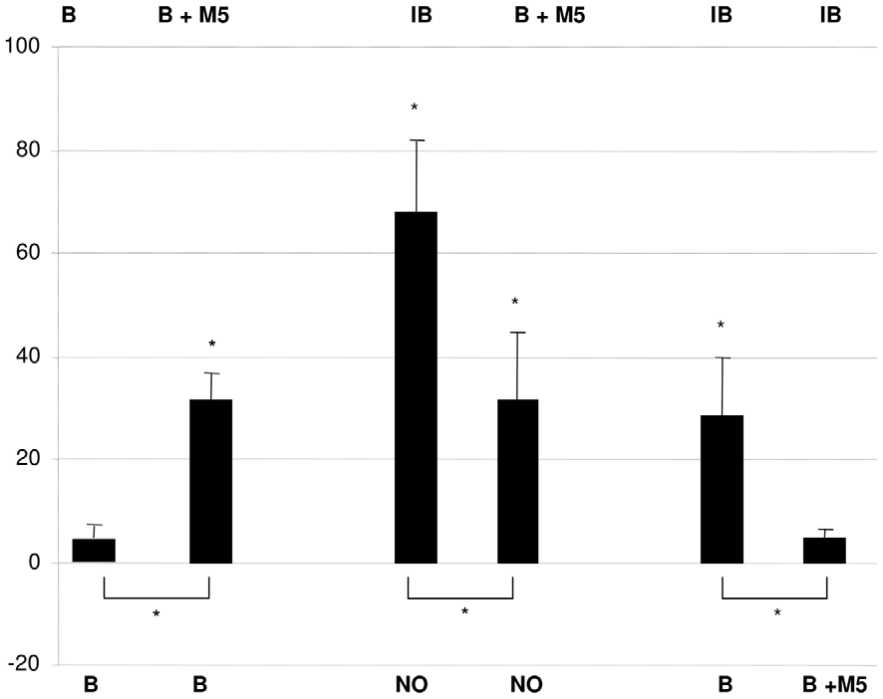
Response to a mixture of all five spider mite induced volatiles of Lima bean. The odor of spider-mite-infested Lima bean (IB) was more attractive than the artificial mixture, plus a Lima bean leaf disc (B+M5) if no odor (NO) was the alternative. The artificial mixture plus the odor of non-infested Lima bean (B+M5) was preferred over a Lima bean leaf disc (B) to a similar extent as a spider-mite infested leaf disc (IB) was preferred to B. In a direct test the mites did not differentiate between the artificial odor (M5+B) and the odor of spider-mite-infested Lima bean (IB). The Y-axis represents the preference index (−100 total repellence, +100 total attraction). A star above the bar indicates a choice based on significance of G_p_<0.05. Horizontal bracket bars with stars below the bars represent significant differences betwwen the pooled experimental results based on a Chi-square test (P<0.05).

A simple mixture consisting of equal quantities of the five HIPV (MeSA, β-ocimene, cis-3-hexenyl acetate, TMTT and DMNT) in addition to the odor of a Lima bean leaf disc was attractive to *P. persimilis* (G_t df = 6_ = 21.70 P = 0.001, G_p df = 1_ = 18.34 P = 0.000, G_h df = 5_ = 3.35 P = 0.645, Figure 1). Although this mixture was an attractant to the mites, attraction to the natural odor produced by a spider-mite-infested Lima bean leaf-disc was significantly stronger (Χ^2^_df = 1_ = 14.00, P = 10^−4^) if clean air was offered as the alternative (Figure 1). Under natural conditions clean air is not a realistic alternative, however: the predatory mites are much more likely to face a choice between the odor of a spider-mite infested plant and non-infested conspecific plants. Facing this more realistic choice, the predatory mites preferred the artificial mixture over the odor of non-infested Lima bean to an extent similar as they prefer the natural odor of spider-mite-infested Lima bean over the odor of non-infested Lima bean (Figure 1). Moreover, offering the artificial mixture against the natural odor of spider-mite-infested Lima bean revealed that predatory mites did not discriminate between these odors (G_t df = 6_ = 7.91 P = 0.244, G_p df = 1_ = 1.29 P = 0.255, G_h df = 5_ = 6.62 P = 0.250 Figure 1).

### Background odor has an opposite effect on predator attraction to individual HIPV and their mixture

**Figure 2:**
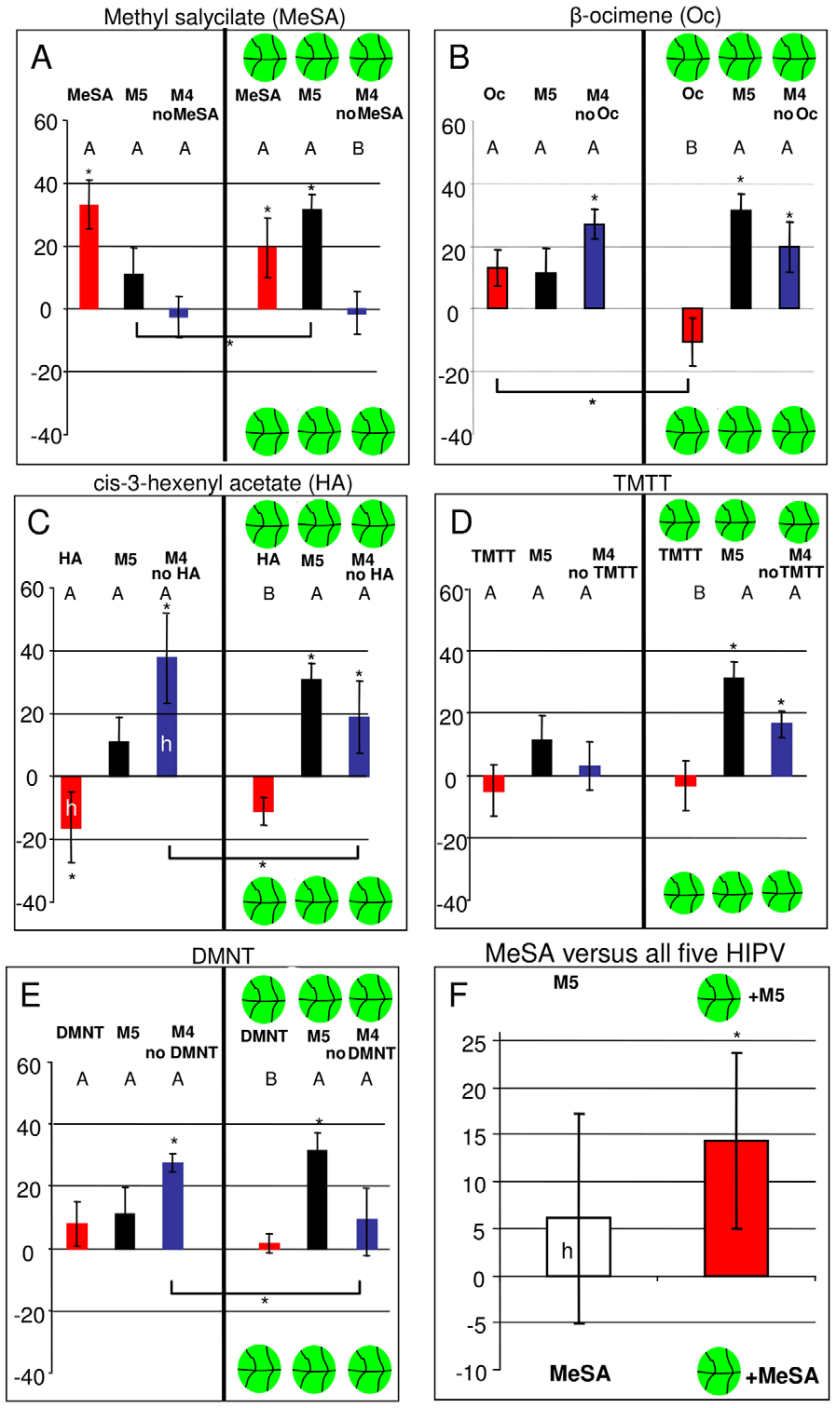
Attraction to individual HIPV, a mixture of all HIPV and mixtures reduced by one component. The left half of each panel depicts results of choice experiments in absence of Lima bean odor while the right half depicts the same experiments in presence of Lima bean background odor (indicated by a leaf disc). The first red bar represents attraction to the pure compound, whereas the second bar represents attraction to the mixture of all five HIPV (for comparison present in all Figures) and the third red bar attraction to a mixture reduced by one HIPV. The Y-axis gives the preference index (−100 total repellence, +100 total attraction). The abbreviation M5 refers to the mixture of all 5 HIPV, and M4 to a mixture reduced by one component. The letter *h* in a bar indicates significant heterogeneity (G_h_<0.05) among replicates. A star above the bar indicates a choice based on significance of G_p_<0.05. Capital letters (A,B) above the bars indicate differences with the mixture (M5) based on ANOVA followed by Dunnett's post-hoc test (P<0.05). Horizontal bracket bars with stars underneath represent significant differences between pooled experimental results, as inferred from Chi-square tests.

When offered in their pure form the five HIPV used to create the attractive artificial mixture elicited responses that ranged from attraction to repellence. MeSA was significantly attractive (G_t df = 6_ = 27.34 P = 0.000, G_p df = 1_ = 21.52 P = 0.000, G_h df = 5_ = 5.81 P = 0.324), whereas attraction to β-ocimene bordered significance G_t df = 6_ = 5.82 P = 0.44, G_p df = 1_ = 3.30 P = 0.070, G_h df = 5_ = 2.605 P = 0.77), TMTT (G_t df = 6_ = 7.182 P = 0.304, G_p df = 1_ = 0.087 P 0.7680, G_h df = 5_ = 7.09 P = 0.214), and DMNT (G_t df = 6_ = 5.40 P = 0.493, G_p df = 1_ = 1.35 P = 0.245, G_h df = 5_ = 0.54 P = 0.542) did not elicit a significant response whereas cis-3-hexenyl acetate was significantly repellent (G_t df = 6_ = 18.25, P = 0.006,G_p df = 1_ = 6.19 P = 0.013, G_h df = 5_ = 12.07 P = 0.034) (Figure 2 A–E). When these odors were tested in the presence of Lima bean background odor the overall effect was that the strength of the response significantly attenuated (F_(1,50)_ = 4.92, P = 0.031) while β-ocimene was the only compound for which attraction significantly decreased (Χ^2^_df = 1_ = 4.40 P = 0.0360). The reverse was true for the blend of these five HIPV. This mixture, presented in background of Lima bean odor elicited a significantly stronger response than in its absence (Χ^2^_df = 1_ = 3.85 P = 0.049). In absence of background odor the mixture of five HIPV was at best a weak attractant (G_t df = 6_ = 10.78 P = 0.090, G_p df = 1_ = 3.57 P = 0.059, G_h df = 5_ = 7.21 P = 0.205). The addition of Lima bean background odor to the HIPV mixture resulted in a more than threefold increase of the preference index and made the mixture a significant attractant (G_t df = 6_ = 21.71 P = 0.001, G_p df = 1_ = 18.34 P = 0.000, G_h df = 5_ = 3.35 P = 0.645). Thus, while the background odor significantly attenuated the response to the components of the artificial mixture, it greatly facilitated attraction to their mixture.

### The relation between the response to HIPV in their pure form and their role in the mixture

MeSA was the only attractive HIPV out of the five that are part of the artificial mixture and mixtures without MeSA were not attractive. There was however no significant difference between attraction to the full mixture of five HIPV and the mixture reduced by eliminating MeSA. However, in the presence of background odor of Lima bean a significant difference between the attraction to the mixture without MeSA and the full mixture was observed (Χ^2^_df = 1_ = 10.99, P = 0.001 Figure 2A).

Removing the most repellent HIPV, cis-3-hexenyl acetate, resulted in increased attraction to the mixture in absence of background odor (Figure 2C). The response to this reduced mixture was significantly heterogeneous, however (G_h df = 5_ = 20.54 P = 0.001). The difference between this reduced mixture and the full mixture bordered significance (Dunnett's post hoc test P = 0.08). In the presence of the background odor, cis-3-hexenyl acetate did not significantly repel the predatory mites and removing it from the mixture had no significant effect either.

TMTT and DMNT elicited no response in the presence, and in the absence of the background odor (Figure 2D,E). The mixture lost its attractiveness, however, when DMNT was eliminated from it, whereas eliminating TMTT from the mixture had no effect.

Attraction to pure β-ocimene was significantly lower in presence, than in absence of background odor (Χ^2^_df = 1_ = 4.49, P = 0.036 Figure 2B). Attraction to mixtures without β-ocimene were not significantly different from attraction to the full mixture. In absence of background odor the reduced mixture was however significantly attractive to the mites, in contrast to the full mixture (G_t df = 6_ = 14.32 P = 0.026, G_p df = 1_ = 7.97 P = 0.004, G_h df = 5_ = 6.35 P = 0.273).

### Background odor facilitates discrimination between MeSA and the HIPV mixture

Starved females of *P. persimilis* cultured on spider-mite-infested Lima bean were attracted to pure MeSA. MeSA was an ingredient of all odor mixtures that significantly attracted *P. persimilis*. In the presence of the background odor of Lima bean there was no difference between the attraction to MeSA and the mixture of all five HIPV. To test if the mites discriminate between MeSA and this mixture these odors were offered as alternatives. Whereas pure MeSA offered against clean air was significantly more attractive than the mixture of all five HIPV offered against clean air (Χ^2^_df = 1_ = 3.5, P = 0.049 Figure 2A) the mites did not prefer MeSA over the mixture when offered as alternatives (G_t df = 6_ = 13.32 P = 0.038, G_p df = 1_ = 0.73 P = 0. 394, G_h df = 5_ = 12.59 P = 0.027 Figure 2F). When the odor of Lima bean was added to both sides of the test arena, the predatory mites preferred the odor of the full mixture over MeSA (G_t df = 6_ = 12.65 P = 0.048, G_p df = 1_ = 3.73 P = 0.053, G_h df = 5_ = 8.92 P = 0.112).

## Discussion

To elucidate if predators respond to the odor of herbivore-infested plants as a whole, we will first assess how important the relative abundance of herbivore-induced plant volatiles is to predator attraction. Then, we will consider to what extent attractive components affect the response to odor mixtures and finally to what extent non-induced plant odors affect predator attraction.

The predatory mites in our experiments preferred the odor of a freshly excised spider-mite-infested leaf disc over the odor of a control leaf disc. The presence of volatiles emanating from the wounded plant tissue did not hamper the predator's ability to recognize the HIPV producing spider-mite-infested odor source. Although there exists a clear difference in the relative abundance of HIPV in the artificial mixture (i.e. equal quantities of each volatile) and the odor of spider-mite-infested Lima bean [22], [23], the mites were not able to discriminate between the two. These results indicate that HIPV are crucial for attraction while their relative abundance appears to play a minor role.

Methyl salicylate was the only HIPV attractive in its pure form and was present in all attractive mixtures. Methyl salicylate is induced by *T. urticae* in a wide variety of plants [24]. It is however by no means a spider-mite specific signal, it is for example also induced by mechanical damage similar to that caused by chewing of a caterpillar [25]. Females of *P. persimilis* cultured on MeSA emitting plants are attracted to pure MeSA [11], [15], [19], [20], [26] whereas without prior experience with MeSA-containing odors in association with prey they are not [15]. Predators easily acquire a preference for odors without MeSA over MeSA containing odors [17], [19]. As far as the population mean response is concerned attraction to MeSA is thus acquired and not innate.

Acquired attraction to pure MeSA either results from the mite's ability to detect and associate the component MeSA in prey-associated odors or from a perceptual similarity between MeSA and these MeSA containing odors. Detection of MeSA in complex mixtures requires that odors are perceived as elemental objects. If attraction to pure MeSA results from the mite's ability to detect this attractant in complex mixtures, all MeSA containing odors assessed in our experiments should be attractive. There were however several mixtures that contained MeSA while they were not attractive: (1) in absence of the background odor the mixture of all five HIPV and (2) the reduced mixture without TMTT, (3) in the presence of the background odor the reduced mixture without DMNT. Since pure MeSA is attractive to *P. persimilis*, this suggests that the mites are not able to detect MeSA in these odor mixtures. Moreover, none of the other HIPV are attractive in their pure form indicating that these components are also not associated with the presence of prey.

It thus appears that predatory mites do not rely on the detection of a single attractant in odor mixtures. Results also indicate that there is no special combination of HIPV that acts as an attractant because the combination of all HIPV is not an attractant while MeSA alone is. This suggests that the MeSA containing odors that were not attractive are to the predators perceptionally dissimilar from MeSA and all attractive MeSA containing odors. To further investigate this question one could perform a cross-generalization experiment to assess if predatory mites cultured in the presence of one of the MeSA containing odors that was not attractive in our experiments acquire a preference for this odor and subsequently ask if the mites are no longer attracted to pure MeSA or any of the attractive MeSA containing odors. Results of such experiments are however difficult to interpret since cross-generalization is often asymmetrical, i.e. if there is generalization from odor a to b there might not be (equal) generalization from odor b to a [27].

Attraction to the following HIPV (mixtures) was significantly affected by the presence of odors not induced by herbivory: (1) the blend of all five HIPV, (2) β-ocimene, (3) the artificial mixture reduced by cis-3-hexenyl acetate and (4) the artificial mixture reduced by DMNT. If the mites perceived these HIPV (mixtures) as components, regardless of the presence of odor not induced by herbivory, experiments with or without it should yield the same result because constitutive plant odor was offered at both sides of the choice arena. Therefore, this is to our knowledge the first unambiguously result showing that constitutive plant odor affects the response to HIPV. This is consistent with the hypothesis that odors are perceived as a whole but not with the hypothesis that predators are attracted to (components of) HIPV.

The idea that the predators perceive odor mixtures as a synthetic whole is further corroborated by the fact that components which in their pure form elicit no response contribute to the response elicited by mixtures they are part of. This phenomenon was also observed in experiments where the response of *P. persimilis* to binary odor mixtures was assessed [21]. In the present study this is best exemplified by the observation that there is no attraction to the reduced mixture without DMNT in presence of background odor (Figure 2E). This suggests that this reduced mixture is perceptually so different from the odor of spider-mite-infested Lima bean that the mites fail to recognize the reduced mixture as a similar odor.

We observed two seemingly opposite phenomena. The predatory mites robustly generalized their response to several odors that are similar to the odor of spider-mite-infested Lima bean. The mites did not differentiate between our artificial mixture and the odor of spider-mite-infested Lima bean even though both odors have very different HIPV ratios, the mites were not troubled by volatiles emanating from leaf disc edges, several HIPV could be removed from the mixture without an apparent effect the mixture's attractiveness and reducing the odor mixture to MeSA resulted in attraction. At the same time the mites did not generalize their response to several odors that were chemically not very different from the full blend such as the mixture without DMNT or the mixture without MeSA in presence of the background odor. How can the lack of response to this chemical variation be reconciled with the responsiveness to other variation? It has been suggested that the glomerular olfactory bulb of vertebrates - a similar system is present in predatory mites [28], [29] - functions as a classification system [30]. If one slowly modifies a binary mixture of two components from a ratio where the first is abundant to a ratio where the later is abundant, the activity of the output neurons of this system remains in one correlated state up to a point, where the output activity suddenly changes and assumes a different state [30]. Each state of the olfactory bulb output neurons is thought to represent class of odors that are perceived as similar [30]. Hence, a range of changes to in the relative abundance of components in odor mixtures may have little or no perceived effect while a small change, even one in the same direction, may suddenly induce a state shift in the bulbar output resulting in a large effect on perception. If we assume that the glomerular olfactory bulb of mites performs the same classification task as its vertebrate analogue and we consider that the resolving power of such a system in mites is constrained by the small number of glomeruli and neurons in mites [28] compared to vertebrates we have a plausible mechanism that can explain our results without invoking a role of components as attractants. A coarse classification system with sudden transitions between odor classes is consistent with low sensitivity to the relative abundance of HIPV and the similarity of a mixture to one component (MeSA) but not to other components. It may explain the observed ability to generalize from a familiar, spider-mite-infested-plant odor to a variety of spider-mite infested plants from different species [24] or to plants infested with prey and non-prey herbivores [31]. This mechanism is also consistent with the observation that not all mixtures that contain an attractive dose of MeSA are attractive to *P. persimilis* and that components that elicit no behavioural response or components that are not induced by the prey contribute to the attractiveness of mixtures they are part of.

Our results suggest that it may be wrong to think of herbivore-infested plant odors as a collection of attractive and repellent components. It appears that the mites like so many other animals [3], [4], [5], [6], [7] experience an odor mixture as a distinct odor, different from its components. Attraction to components of a mixture may arise from perceptual similarity to the mixture but this does not necessarily imply that the presence of such components in other mixtures makes these (more) attractive.

## Materials and Methods

### Plants and Mites

Lima bean plants (*Phaseolus lunatus*) were grown in a climate room (22°C, 60% RH, 16∶8 LD) until they were two weeks old. Subsequently, the plants were infested with two-spotted spider mites *Tetranychus urticae* (Koch). Predatory mites (*Phytoseiulus persimilis* Athias-Henriot) were reared in a climate room (25°C, 80% RH, 16∶8 LD) on detached spider-mite-infested Lima bean leaves. Every day predatory mites received fresh spider-mite-infested Lima bean leaves and the culture was subject to harvesting virtually every working day. The frequent harvesting of mites ensured that most mites used in the experiments were one to a few days old since their last moult in the adult phase. Predatory mites were originally obtained in 2001 from field samples at various sites, where they naturally occur near the coast of Sicily, Italy. Before choice tests female predatory mites were taken from the culture and kept in Eppendorf tubes, deprived of water and food for a period of 16–22 hours.

### Odors

Methyl salicylate (MeSA) was obtained from Sigma-Aldrich, β-ocimene (70% E- and 30% Z- isomers) from R. C. Treatt & co, cis-3-hexenyl acetate was obtained from Aldrich, (E)-4,8-dimethyl-1,3,7-nonatriene (DMNT) and (E,E)-4,8,12-trimethyl-1,3,7,11-tridecatetraene, (TMTT) were generously provided by Dr. W. Boland of the Max Planck Institute for Chemical Ecology, Jena, Germany. The concentration of each individual compound and each compound in a mixture consisted of a 1∶10,000 dilution of the odor in hexane. Small pieces of filter paper (Ø 0.5 cm divided in two pieces) were provided with 0.5 µl of the odor solutions and served as the odor sources in the choice experiment. Freshly excised leaf discs (Ø 1 cm) of non-infested Lima bean served as the background odor and could easily be provided with pieces of filter paper containing different combinations of HIPV. Freshly excised leaf discs and freshly made odor sources were used in each replicate experiment. In a series of choice experiments the preference for: (1) each of the five HIPV (MeSA, β-ocimene, cis-3-hexenyl acetate, DMNT and TMTT) was tested against clean air, (2) each of these five HIPV added to odor from a leaf disc was tested against odor from a leaf disc, (3) a mixture of four out of the five HIPV was tested against clean air, (4) a mixture of four of the five HIPV added to odor from a leaf disc was tested against odor from a leaf disc, (5) the full mixture of all five HIPV was tested against clean air, (6) the full mixture of all five HIPV added to a leaf disc was tested against odor from a leaf disc. Finally, the preference for the full mixture of these five HIPV was tested against MeSA, either with or without a background odor from a herbivore-free leaf disc at either of the two alternative odor sources.

### Olfactory response tests

**Figure 3:**
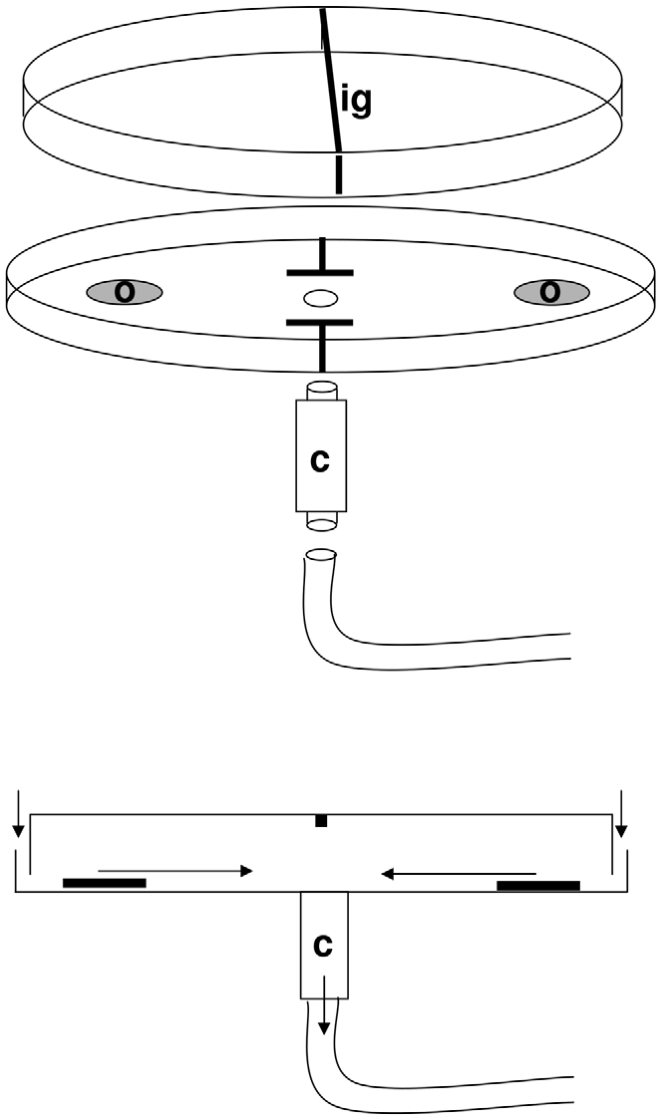
The experimental setup. The choice arena was constructed from a Petri dish (Ø 9 cm) positioned up side down. An insect glue barrier (ig) divided the dish in two compartments that each contained an odor source (o) An opening at the bottom allowed for the connection of a cartridge containing the mites. The cartridge (c) was fitted to a vacuum pump. The vacuum gives rise to a radial airflow over the bottom of the choice arena, thus establishing two odor fields that extended from the odor sources to the cartridge. Arrows indicate air flow direction in the system.

The choice tests were conducted as described in [19], [21]. In short, the response to the odors was assessed using an experimental arena, constructed from a Petri dish (Ø 9 cm) put upside-down (Figure 3). A radial airflow was established by the connection of a vacuum pump (flow 0.42 l/min) to an opening at the centre of the bottom of the arena. Prior to the experiment, groups of about 35 mites were placed in cartridges that could be fitted between the vacuum pump and the centre of the arena. For each replicate experiment the setup was provided with freshly prepared odor sources and a new cartridge with a new group of predatory mites. To avoid contamination, different odors were tested in a different arena. To confine the mites in the segment containing the odor of their choice, insect glue barriers divided the arena in two while leaving a 3 cm glue-free space in the middle on the arena bottom. In this way, the mites were allowed to move only from the cartridge to either segment, or back and forth between both segments via the 3 cm wide opening in the insect glue barrier. One side contained the synthetic odors and the other contained a control filter paper impregnated with the solvent only. If Lima bean background odor was provided, both sides contained a herbivore-free Lima bean leaf disc and one side additionally contained a filter paper with the synthetic odor while the other side contained the control filter paper with solvent. The odor sources were prepared in a fume hut and the solvent was allowed to evaporate for exactly one minute before the odor source was placed in the arena. The mites were released from the cartridge and after three minutes the mites at each side of the choice dish were counted.

For graphical display of the results, the preference for odors over the control is expressed as a preference index: ((mites at odor side – mites at control side)/total amount of mites) * 100. In this way aversive odors were assigned a negative preference index (−100 to 0) and attractive odors a positive preference index (0 to 100).

### Statistics

Because there is no difference between the results obtained from mites tested individually and mites tested in groups in the two-choice test employed [21], we can assume that individual mites make individual choices. A replicated G-test for goodness of fit [32] was used to assess if compounds elicited a response; significant values of the total G-statistic (G_t_) indicate a deviation from the expected binomial distribution around a 50% response. This statistic can be broken down into two statistics that each indicate different aspects of the deviation. The overall pooled deviation from an even distribution of all 6 replicate experiments is indicated by significant values of the pooled G statistic (G_p_) while the second statistic, G_h_, indicates the degree of heterogeneity among the 6 replicate experiments.

Individual synthetic compounds and their mixtures were compared to the full mixture of five compounds using ANOVA followed by Dunnet's post hoc test [33]. This analysis was performed on the (arcsine square root) transformed, relative frequencies of replicate experiments [32]. If replicate experiments were not heterogeneous, (indicated with an *h* in the bars of the Figures), replicate experiments were pooled and the grand totals of mite choices can be used to compare treatments in 2×2 frequency tables using a Chi square test.
